# Supplementary material for: Exploratory Analysis of Selected Components of the mTOR Pathway Reveals Potentially Crucial Associations with Childhood Malnutrition
Source: Nutrients. 2022 Apr 12;14(8):1612. doi: 10.3390/nu14081612 (PMC9031007; doi:10.3390/nu14081612)
Supplement: Supplementary file 1 [file nutrients-14-01612-s001.zip › nutrients-1628419-supplementary.pdf]

Table S1. Comparison of OD values for phospho-mTORC1 at 450 nm between each of the three malnourished study groups with that obtained from the healthy control group.

OD values for phospho-mTORC 1	Stunted vs. Healthy Control			At risk of stunting vs. Healthy Control			SAM vs. Healthy Control		
	Stunted	Healthy Control	p-value	At risk of stunting	Healthy Control	p-value	SAM	Healthy Control	p-value
	0.33 (0.22-0.43)	0.41 (0.31-0.50)	0.13	0.33 (0.18-0.48)	0.41 (0.31-0.450)	0.46	0.25 (0.12-0.38)	0.41 (0.31-0.450)	0.011*

[1] Data expressed as median and Q1-Q3; [2] *p*-value shown was a result of Mann-Whitney U-test involving the comparison of OD values for phosphor-mTORC1 at 450 nm between each of the three malnourished study groups with that obtained from the healthy control group. Results with p value of less than 0.05 have been marked (*) as statistically significant.

Table S2. Results of bivariate logistic regression for each of the continuous and categorical variables between each of the three malnourished study groups and the healthy control group.

Continuous Variables	Stunted vs. Healthy Control		At risk of stunting vs. Healthy Control		SAM vs. Healthy Control	
	Odds ratio (95% CI)	p-value	Odds ratio (95% CI)	p-value	Odds ratio (95% CI)	p-value
Maternal age	0.91 (0.81-1.02)	0.11*	0.97 (0.88-1.08)	0.60	0.97 (0.88-1.07)	0.56
Paternal age	0.95 (0.87-1.03)	0.19*	1.0 (0.93-1.09)	0.88	0.98 (0.9-1.06)	0.55
Working mother	2.07 (0.18-24.1)	0.56	5.77 (0.63-52.6)	0.12*	2.07 (0.18-24.1)	0.56
Duration of EBF	1.02 (0.85-1.23)	0.83	0.93 (0.77-1.13)	0.46	0.98 (0.81-1.19)	0.85
Currently breastfed	1.55 (0.24-10.0)	0.64	1.0 (0.13-7.59)	1.0	5.93 (1.16-30.3)	0.032*
Currently formula fed	1.69 (0.62-4.63)	0.31	1.48 (0.54-4.10)	0.44	0.11 (0.02-0.54)	0.007*
Currently fed with rice powder or suji	1.33 (0.47-3.82)	0.59	1.09 (0.39-3.11)	0.81	0.13 (0.03-0.52)	0.004
Currently on cow/goat's milk	0.59 (0.18-1.91)	0.38	0.69 (0.21-2.29)	0.55	0.20 (0.06-0.62)	0.005*

Table S3. Primer sequences for the genes used for the gene expression analyses.

Gene	Forward Primer	Reverse Primer
MTOR	GCTTGATTTGGTTCCCAGGACAGT	GTGCTGAGTTTGCTGTACCCATGT
TSC1	GCAGCGTGACACTATGGTAACCAA	AGTTCTATCCGCAGCTCCGCAAT
LAMTOR2	TCGGAGATCTGGGTGCAAAA	GTCAGTGTCCCCGTAACCCAG
RPS6K1	ACTGTAGTGTTGACTGCCTGACCA	TAGCCAGCCATCACAGTGCTCAT
RICTOR	GGAAGCCTGTTGATGGTGAT	GGCAGCCTGTTTTATGGTGT
GAPDH	GAGTCAACGGATTTGGTCGT	GACAAGCTTCCCGTTCTCCAG
